# Editorial: Mitochondrial Research: Yeast and Human Cells as Models 2.0

**DOI:** 10.3390/ijms25126344

**Published:** 2024-06-08

**Authors:** Maša Ždralević, Clara Musicco, Sergio Giannattasio

**Affiliations:** 1 Institute for Advanced Studies, University of Montenegro, 81000 Podgorica, Montenegro; masaz@ucg.ac.me; 2 Institute of Biomembranes, Bioenergetics, and Molecular Biotechnologies, National Research Council (CNR), 70126 Bari, Italy; c.musicco@ibiom.cnr.it

Mitochondrial research stands at the forefront of modern biology, unraveling the intricate mechanisms governing cellular metabolism, energy production, and disease pathogenesis. Understanding the elaborate mechanisms governing mitochondrial biology and dysfunction is paramount for the development of novel therapeutic strategies. In this context, the Special Issue “Mitochondrial Research: Yeast and Human Cells as Models 2.0” in the *International Journal of Molecular Sciences* (*IJMS*) emerges as a pivotal platform for advancing our knowledge in this field.

The six open-access papers featured in this Special Issue represent a convergence of interdisciplinary expertise, revealing new insights and methodologies that promise to redefine our understanding of mitochondrial function. This editorial encapsulates the collaborative efforts of researchers in advancing our understanding of mitochondrial biology and dysfunction, paving the way for the development of targeted therapeutic interventions aimed at restoring mitochondrial homeostasis and cellular function.

Mitochondrial proteins orchestrate essential cellular processes, including ATP synthesis and metabolism regulation. The vast majority of mitochondrial proteins are encoded by nuclear DNA and synthesized in the cytoplasm; however, mitochondria themselves are capable of protein synthesis and are specialized for the synthesis of components of the respiratory chain complexes. The regulatory mechanisms governing mitochondrial translation must be very strict in order to enable proper assembly and functioning of the respiratory chain [1]. However, these mechanisms are still not fully understood, and Baleva et al. investigated the role of the mitochondrial steroid receptor RNA activator (SRA) stem–loop-interacting RNA-binding protein (SLIRP) in mitochondrial translation in human embryonic kidney (HEK293T) cells. They showed that *SLIRP* knock-out impaired mitochondrial function and led to a decrease in the protein expression levels of the mitochondrially-encoded CO II and CO III subunits. In addition, the quantity and activity of complexes I and IV were decreased due to the impairment of the translation of their mitochondrially encoded subunits. In fact, SLIRP was shown to interact with the small subunit of the mitochondrial ribosome, thereby possibly interfering with the initiation of mitochondrial translation.

Bhat et al. presented a rigorous new technique for measuring mitochondrial function in individual cells based on the activity of succinate dehydrogenase (SDH), which was determined using confocal microscopy in human airway smooth muscle (hASM) cells. By performing quantitative histochemical analysis of SDH activity in individual hASM cells, the researchers were able to provide a reliable and reproducible estimate of the maximal respiratory capacity of these cells, which can be used in addition to established respirometry-based oxygen consumption rate measurements.

Understanding the effects of viral infection on mitochondrial morphology and function is of great importance, in particular regarding their involvement in immune response mechanisms [2]. Replication of positive-strand RNA viruses is known to occur in association with host cell membranes. Carnation Italian Ring-spot Virus (CIRV) is an important model plus-strand RNA virus, and Petrosillo et al. investigated the effects of CIRV p36 ectopic expression on mitochondrial dynamics and respiratory chain complex function in *Saccharomyces cerevisiae* yeast. p36 is required for targeting the virus replication complex to the outer mitochondrial membrane [3]. In the yeast model used by Petrosillo et al., p36 expression was shown to induce alterations in mitochondrial network, which was associated with a decrease in mitochondrial respiration and the activities of respiratory complexes C II–III and C IV. This study sheds light on virus–host interactions at the mitochondrial level, providing insights into viral pathogenesis and host defense mechanisms. Understanding these interactions is crucial for developing antiviral strategies and advancing our knowledge of mitochondrial biology.

Beyond their essential role in metabolism and energy production, mitochondria are pivotal players in regulated cell death as well [4]. Apoptosis, as a form of regulated cell death, is controlled by proteins of BCL-2 family, which modulate mitochondrial outer membrane permeabilization and can have either pro- or anti-apoptotic activities [5]. Mentel et al. investigated the role of the yeast Bax inhibitor Bxi1p/Ybh3p on the pro- and anti-apoptotic roles of BCL-2 family members, as well as survival after treatment with inducers of regulated cell death, using yeast *S. cerevisiae* as a model. The activity of BCL-2 family members, including pro-apoptotic Bax and Bak and anti-apoptotic Bcl-XL and Bcl-2, was not affected by *BXI1* deletion, although it resulted in increased sensitivity to acetic acid.

Mitochondrial metabolism in yeast is highly influenced both by the type of carbon source (fermentative or respiratory) [6] and by the activity of signaling pathways, such as cAMP-dependent protein kinase A (PKA) and TOR signaling [7]. It is also tightly coupled with cell cycle progression. Leite et al. reported on the role of the anaphase-promoting complex/cyclosome (APC/C) activator Cdh1p in mitochondrial metabolic remodeling in yeast. APC/C is an E3 ubiquitin ligase that regulates the proteolysis of many cell cycle regulators, and is itself primarily regulated by the activity of Cdh1p and Cdc20p cofactors [8]. Cdh1 was shown to be implicated in mitochondrial metabolism, as well as to affect mitochondrial oxygen consumption and cytochrome c oxidase activity, mediated by the transcriptional activator Yap1, a key regulator of the yeast oxidative stress response. Therefore, this study helps in elucidating the interplay between cell cycle regulation and mitochondrial metabolism, contributing to our understanding of the signaling pathways controlling energy homeostasis.

Finally, Bruni provided an excellent review of human long non-coding RNAs (ncRNAs) encoded by the mitochondrial DNA (mt-lncRNAs). Mt-lncRNAs were shown to have disparate cellular localizations and functions; they are found not only in mitochondria, but also in the nucleus, nucleolus, cytoplasm, and exosome, and are found to be involved in the regulation of mitochondrial gene expression, translation, cell proliferation, retrograde signaling, immune response, and as structural components of mitochondrial ribosomes. In addition, mt-lncRNAs are increasingly being recognized as significant contributors to human diseases, such as cancer and neurodegenerative diseases. Consequently, unraveling the functions of mt-lncRNAs could lead to new therapeutic avenues for mitochondrial disorders and other diseases.

In conclusion, the papers featured in this Special Issue represent a distinct set of cutting-edge contributions to the field of mitochondrial research, leveraging yeast and human cell models to unravel the complexities of mitochondrial biology and dysfunction. As we continue to decipher the molecular intricacies governing mitochondrial function, these findings serve as a foundation for the development of targeted therapeutic strategies aimed at mitigating mitochondrial dysfunction and restoring cellular homeostasis.
